# The role of mononuclear phagocyte system in IgA nephropathy: pathogenesis and prognosis

**DOI:** 10.3389/fimmu.2023.1192941

**Published:** 2023-07-03

**Authors:** Yiwen Liu, Yan Gong, Gaosi Xu

**Affiliations:** ^1^ Department of Nephrology, The Second Affiliated Hospital of Nanchang University, Nanchang, Jiangxi, China; ^2^ The Second Clinical Medical College of Nanchang University, Nanchang, Jiangxi, China; ^3^ Department of Neurosurgery, The Second Affiliated Hospital of Nanchang University, Nanchang, Jiangxi, China

**Keywords:** IgA nephrology, mononuclear phagocyte system, M1 macrophage, M2 macrophage, pathogenesis

## Abstract

Although the “multiple hits” theory is a widely accepted pathogenesis in IgA nephropathy (IgAN), increasing evidence suggests that the mononuclear/macrophage system plays important roles in the progression of IgAN; however, the exact mechanism is unclear. In the present study, we explored 1,067 patients in 15 studies and found that the number of macrophages per glomerulus was positively related with the degree of hematuria, and the macrophages in the glomeruli were mainly related to mesangial proliferation (M) in renal biopsy. In the tubulointerstitium, macrophages were significantly paralleled to tubulointerstitial α-SMA and NF-kB expression, tubulointerstitial lesion, tubule atrophy/interstitial fibrosis (T), and segmental glomerulosclerosis (S). In the glomeruli and tubulointerstitium, M1 accounted for 85.41% in the M classification according to the Oxford MEST-C, while in the blood, M1 accounted for 100%, and the patients with low CD89^+^ monocyte mean fluorescence intensity displayed more severe pathological characteristics (S1 and T1-2) and clinical symptoms. M1 (CD80^+^) macrophages were associated with proinflammation in the acute phase; however, M2 (CD163^+^) macrophages participated in tissue repair and remodeling, which correlated with chronic inflammation. In the glomeruli, M2 macrophages activated glomerular matrix expansion by secreting cytokines such as IL-10 and tumor necrosis factor-β (TGF-β), and M0 (CD68^+^) macrophages stimulated glomerular hypercellularity. In the tubulointerstitium, M2 macrophages played pivotal roles in renal fibrosis and sclerosis. It is assumed that macrophages acted as antigen-presenting cells to activate T cells and released diverse cytokines to stimulate an inflammatory response. Macrophages infiltrating glomeruli destroy the integrity of podocytes through the mesangio-podocytic-tubular crosstalk as well as the injury of the tubule.

## 1 Introduction

IgA nephropathy (IgAN) is a primary glomerular disease worldwide and is characterized by the deposition of IgA1 immune complexes in the glomerulus region with mesangial hyperplasia and mesangial matrix expansion (1–4). In China, IgAN is the significant reason for end-stage renal disease (ESRD), and up to 15% to 20% of IgAN patients will develop chronic kidney injury within 10 years after being diagnosed (5, 6).

At present, the “multiple hits” theory is widely accepted (7). Increased research suggested that the mononuclear/macrophage system played pivotal roles in the progression of IgAN. The infiltration of macrophages is associated with the release of proinflammatory cytokines and chemokines, which have been regarded as significant components in the pathogenesis of renal disease (8–10). In the glomeruli, the number of CD68^+^ macrophages is higher than CD206^+^ macrophages in IgAN. However, in the tubulointerstitium, the number of CD68^+^ macrophages is similar to CD206^+^ macrophages (11), which means that there are various subclasses and a number of macrophages infiltrating different parts of the kidney. Macrophages infiltrating the glomeruli are paralleled to the severity of hematuria and crescent formation, while macrophages infiltrating the tubulointerstitial department enhance the degree of 24-h urinary protein quantification, renal tubules, and interstitial lesion (12–14). The intensity of glomerular macrophage infiltration is regarded as the predictor for the probability of response to immunosuppressive therapy (11).

Nevertheless, the relationships between the number, subtype, and invasion site of macrophages and the mechanisms, clinical manifestations, and prognosis in IgAN remain unclear (15). The present study explored the different characteristics of macrophages in IgAN found in the glomerulus compared to the tubulointerstitium and related those characteristics to clinical outcomes and also explored the roles of the mononuclear/macrophage system in the pathogenesis and the prognosis in IgAN.

## 2 The macrophage subpopulations and their functions in IgAN

Macrophages are known as heterogeneous populations, which are subdivided into different groups based on their surface molecules and functions (16). In IgAN, these groups of macrophages might exist differently and have diverse effects on the renal injury of IgAN (17). M1 macrophages are correlated to proinflammatory reactions in the acute phase, during the first 48 h after an acute kidney injury (AKI), while M2 macrophages, which are relevant to chronic inflammation, take part in tissue repair and remodeling and dominate at later time points (18, 19). M2 macrophages are subdivided into three subclasses. M2a macrophages are associated with wound healing marked by CD206, M2b macrophages can take part in the immune response marked by CD86, and M2c macrophages are regulatory cells marked by CD163 (20). Light microscopy displays all kinds of morphological variations, such as mesangial matrix expansion, mesangial or endocapillary proliferation, and fibrosis or crescent formation. During the chronic phase, global or segmental glomerular sclerosis, renal tubular atrophy, and tubulointerstitial fibrosis are seen (21).

**Figure 1:**
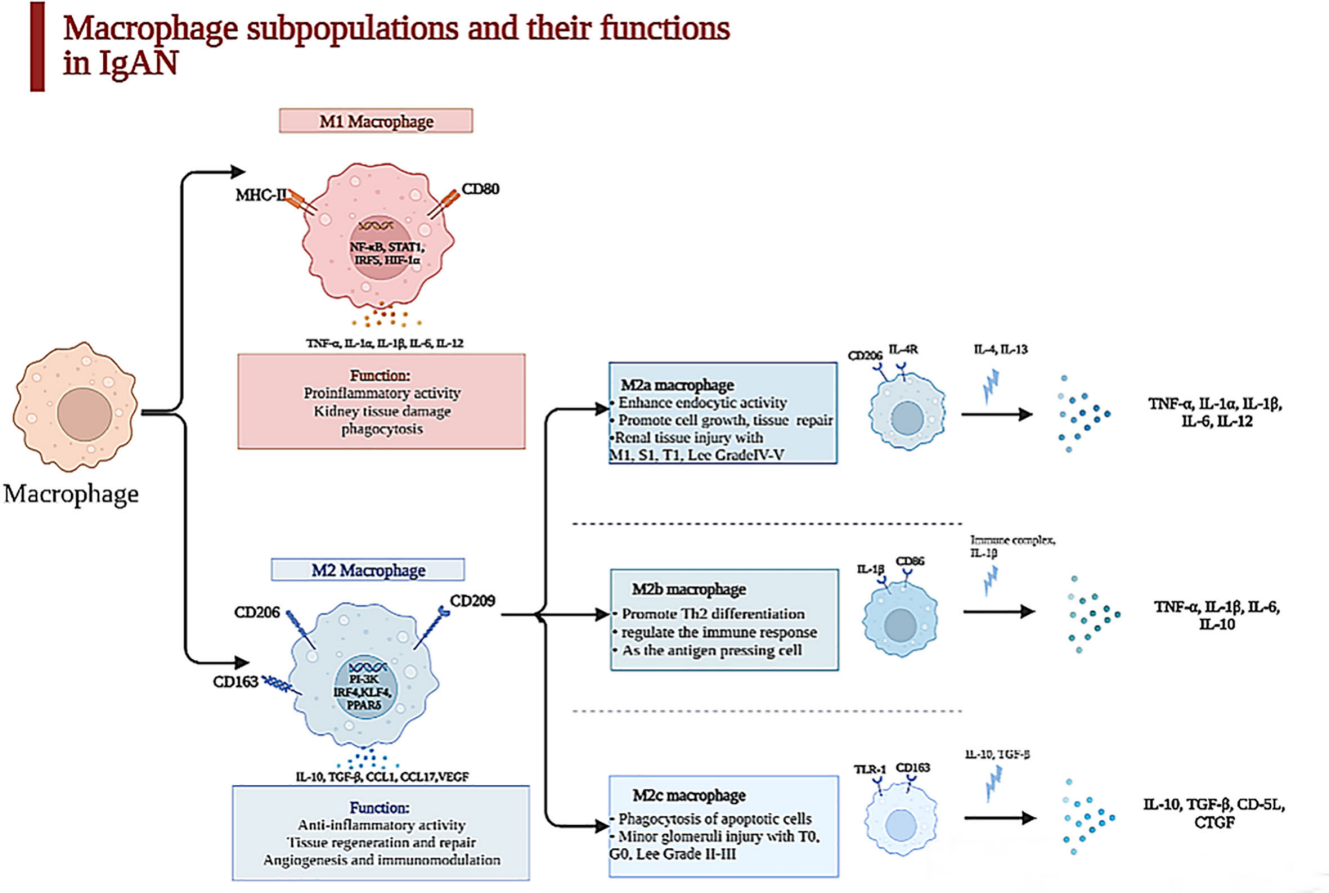
Macrophage subpopulations and their functions in kidney. Macrophages were heterogeneous populations, which were divided into two subpopulations in kidney. The surface marker of M1 macrophages is CD86 which is characterized by a proinflammatory phenotype in acute phase during the first 48 h after an acute injury through secreting TNF-α, IL-6, IL-1β, etc., which can lead to kidney tissue damage. In contrast, M2 macrophage is CD163, which is relevant to chronic inflammation, taking part in tissue repair and remodeling and dominating at later time points in IgAN. With the stimulation of IL-4 and IL-13, M2a macrophages can promote cell growth and tissue repair and are paralleled to renal tissue injury with M1, S1, T1, and Lee grade IV–V secreted TNF-α, IL-1β, IL-6, etc. M2b enhances crescent formation and activates T cell as the antigen-presenting cell, but M2c is related to minor glomerulus injury with T0, G0, and Lee grade II–III.

The quantity and the subtype of macrophages are correlated with the clinical manifestations and pathology of IgAN patients. M0 macrophages are polarized to M1 macrophages through the nuclear factor-κB (NF-κB) pathway after TNF-α and IFN-γ stimulation, and M1 macrophages recruited to glomeruli can facilitate inflammatory response and process of mesangial fibrosis, but the polarization of M2 macrophages is stimulated through the PI3K pathway after the activation of IL-10, TGF-β, etc., and they can secrete cytokines, which repair renal damage by accelerating proliferation of mesangial cells and interstitial fibrosis and stimulating angiogenesis (22, 23). M2a macrophages stimulated by IL-4 or IL-13 are related with renal repair and the process of kidney fibrosis; M2b macrophages, which can modulate the immune response, are activated by IL-1β; M2c macrophages induced by IL-10 and TGF-β have the functions of profibrosis and anti-inflammation (20), as displayed in **Figure 1**.

## 3 The role of mononuclear phagocyte system in glomeruli

### 3.1 The relationship between different subtypes of macrophages and clinical characteristics

Infiltration of macrophages is a universal feature in IgAN patients with glomerular injury. We summarized the clinical and pathological characteristics of IgAN patients with the infiltration of macrophages in the glomeruli, as demonstrated in **Table 1**. There were 12 articles on IgAN concerning the infiltration of macrophages in the glomeruli, and 87.5% of the patients belonged to the M1 according to the Oxford MEST-C. Hematuria and proteinuria are usual symptoms in IgAN patients. Kawasaki et al. showed the connection between the clinical manifestations of 41 patients with the infiltration of macrophages by renal biopsy (17). The average number of glomerular macrophages per patient (ANM/P) is linked with the severity of hematuria and leukocyturia positively (*p* < 0.01) (29), and the macrophage subtypes were associated with pathological subclasses according to MEST-C. CD68^+^ macrophage counts per glomerulus were related with C (r = 0.346), M (r = 0.325), and final MEST-C sum scores (r = 0.337) (12). CD163^+^ macrophages in the glomeruli were pertinent to the estimated glomerular filtration rate (eGFR) (r = −0.523) and crescents (r = 0.730) (18).

**Table 1:** Summary references of mononuclear phagocyte system in IgA nephropathy.

Author	Year	Age	Sex (M/F)	Renal biopsy	Blood test	Urinalysis	Hypertension	Infiltration of mononuclear macrophages	Outcome	Conclusion
Glomeruli and tubulointerstitium										
Caliskan Y (12)	2022	37 ± 13	31/16	M1: 26 E1: 16 S1: 27 T0: 32 T1: 14 C0: 25 C1: 11	Scr (mg/dl) median (IQR): 1.2 (1.0–1.6) ALB (g/L) median (IQR): 42 (36–44) eGFR (ml/min/1.73 m^2^): 70.3 ± 35.6 Serum Gd-IgA1 (µg/µl) median (IQR): 6.18 (3.16–12.44) Serum AOPP (µmol/L) median (IQR): 90 (47–169)	Macroscopic hematuria, n = 5 Microscopic hematuria, n = 30 Proteinuria (g/day) median (IQR): 1.9 (1.0–4.0)	Systolic BP (mmHg): 131 ± 14 Diastolic BP: 78 ± 9 Hypertension presentation: n = 15	CD68^+^ cell count (glomerular) median (IQR): 4.4 (2.8–11.1) CD68^+^ cell count (tubulointerstitium) median (IQR): 10 (5–30)	The number of CD68^+^ cells per glomerulus is linked to C (r = 0.346), M (r = 0.325), final MEST-C sum scores (r = 0.337) Tubulointerstitial CD68^+^ cell count with T score (r = 0.302) MEST-C score (r = 0.322), proteinuria (r = 0.410)	Macrophages, in the tubulointerstitial compartment, were paralleled to tubular atrophy, interstitial fibrosis, and the degree of proteinuria, while macrophages in glomeruli were related with crescent formation.
Xie D (11)	2021	Responders: 33.5 ± 9.8 Non-responders: 35.5 ± 9.2	Responders: 119/146 Non-responders: 61/92	Responders/non-responders M1: 248/146 E1: 31/14 S1: 227/132 T1: 103/55 T2: 63/61 C1: 115/59	Responders eGFR (ml/min/1.73 m^2^): 85.2 ± 28.9 ALB (g/L): 36.6 ± 6.7 Hemoglobin (g/L): 130.7 ± 20.1 Non-responders eGFR (ml/min/1.73 m^2^): 74.0 ± 30.3 ALB (g/L): 36.7 ± 5.8 Hemoglobin (g/L): 126.4 ± 19.9	Responders 24-h proteinuria (g/day): 1.4 Non-responders 24-h proteinuria (g/day): 1.3	Hypertension presentation (n) Responders: 41 Non-responders: 25	Responders/Non-responders Glomeruli (cells per glomerulus): CD68^+^ macrophages: 4.7/1.5 (*p* < 0.001) CD206^+^ macrophages: 3.3/1.4 (*p* < 0.001) Tubulointerstitium (cells per 100 mm^2^) CD68^+^ macrophages: 2.4/1.9 (*p* = 0.01) CD206^+^ macrophages: 2.4/2.1 (*p* < 0.001)	Macrophages, in the tubulointerstitium positively related with T scores, tubulointerstitial fibrosis, and eGFR attenuation, were the marker of chronic kidney damage.	The quantity of glomerular macrophage can reflect the possibility of reaction to immunosuppression in IgAN patients with a high risk of renal injury.
Yang M (24)	2021	NA	NA	Lee grade III: n = 17 IV: n = 12 V: n = 11	Scr (mmol/L): III: 79.31 ± 19.73 IV: 113.59 ± 24.07 V: 145.40 ± 36.40 eGFR (ml/min/1.73 m^2^) III: 94.59 ± 28.05 IV: 71.18 ± 19.93 V: 49.84 ± 16.41	Proteinuria (g/day) III: 0.92 ± 0.63 IV: 1.50 ± 0.98 V: 3.08 ± 1.62 Urinary RBC (HP) III: 43.47 ± 45.38 IV: 91.49 ± 91.78 V: 88.90 ± 88.59	NA	IgAN patients: Location: macrophages mainly infiltrated the interstitium of the renal tubules and some in the glomerular capillaries and lumen of the renal tubules. M0 macrophages were mainly polarized into M2 macrophages. Control: no distribution	The infiltration of M2 macrophage is related with Scr (r = 0.447) and 24-h proteinuria (r = 0.436) and eGFR (r = −0.332) and fibrotic area (r = 0.777) NS between M0 and M1 and clinical indicator normal	M2 macrophages, AIM, and TGF-β1 play pivotal roles in the course of IgAN fibrosis, which is influenced mutually. M2 macrophage is related with AIM (r = 0.900) and TGF-β1 (r = 0.888). AIM is related with TGF-β1 (r = 0.913).
Hu W (20)	2019	38.76 ± 12.89	22/27	M0: n = 19 E0: n = 48 S0: n = 23 T0/T1: n = 22/27 C0/C1: n = 32/17 Lee grade (n): II: 5 III: 18 IV: 6 V: 13	Scr (μmol/L): 166.19 ± 88.06 ALB (g/L): 37.62 ± 5.67 BUN (mmol/L): 8.96 ± 3.79	Proteinuria (g/day): 1.871 ± 1.865	Systolic BP: 131.88 ± 19.22 Diastolic BP: 81.88 ± 12.70 Hypertensive patients: 55.1%	The numbers of macrophages in the glomerular: Glomerular macrophage: 12.84 ± 7.94 M2a: 7.24 ± 4.39 M2b: 10.72 ± 6.58 M2c: 3.50 ± 1.98 The numbers of macrophages in the tubulointerstitium: Tubulointerstitial macrophage: 26.13 ± 17.68 M2a: 14.47 ± 8.60 M2b: 15.14 ± 12.24 M2c: 6.02 ± 3.68	Macrophages in the tubulointerstitium were related with glomerular sclerosis (r = 0.283), and tubulointerstitial fibrosis (r = 0.400). In tubulointerstitium, the quantities of M2a were positively pertinent to Scr (r = 0.363), 24-h proteinuria (r = 0.329), degree of focal sclerosis (r = 0.457), and tubulointerstitial fibrosis (r = 0.327). M2a macrophages that mainly migrated to tubulointerstitium were related with M1, S1, and T1 in line with the Oxford MEST-C and Lee grade IV–V. The number of M2c macrophages in glomeruli was related with T0, G0, and Lee grade II–III. M2b macrophages were NS.	In IgAN, the counts of M2a and M2b macrophages were higher than those of M2c. M2a macrophages were pertinent to more serious tubulointerstitial pathological features and kidney function, but M2c was negatively associated with glomerular sclerosis and tubulointerstitial fibrosis.
Li J (18)	2015	With crescents: 35 ± 20 Without crescents: 42 ± 10	With crescents: 3/7 Without crescents: 6/8	With/without crescents (mean rank) E: 13.7/12.01 M: 12.55/12.46 S (glomerular): 11.93/12.11 S (focal): 12.65/12.39 T: 12.7/12.36	Scr (g/l) With crescents: 34 ± 7 Without crescents: 41 ± 7 eGFR (ml/min/1.73 m^2^) With crescents: 58 ± 27 Without crescents: 88 ± 22	Proteinuria (g/day) With crescents: 1.8 ± 1.1 Without crescents: 1.0 ± 0.7	NA	CD163^+^ macrophages in tubulointerstitium: With crescents: 26 ± 8 Without crescents: 9 ± 4 CD163^+^ macrophages in glomeruli: With crescents: 4 ± 1 Without crescents: 2 ± 1	CD163^+^ macrophage in tubulointerstitium is related with ALB (r = −0.574), eGFR (r = −0.718), proteinuria (r = 0.520), and with glomeruli crescents (r = 0.821). CD163^+^ macrophage in glomeruli is related with eGFR (r = −0.523) and with glomeruli crescents (r = 0.730).	More CD163^+^ macrophages infiltrated the glomeruli and acute tubulointerstitial lesions in IgAN patients with Formation of crescents.
Ikezumi Y (13)	2011	Children (<10 years): n = 14 Children (> 12 years): n = 15 Adults (26–35 years): n = 27	Children (<10 years): 8/6 Children (>12 years): 6/9 Adults (26–35 years): 11/6	Children (<10 years): Glomerular hypercellularity increased cells in the mesangial and endocapillary cells. Children (>12 years): glomerular lesion more akin to the adult IgAN Adults: glomerular matrix expansion, segmental glomerulosclerosis	eGFR (ml/min/1.73 m^2^): Children (<10 years): 107.4 ± 17.3 Children (>12 years): 121.1 ± 14.1 Adults: 104.3 ± 28.2	Proteinuria (g) (1.73 m^2^ /day): Children (<10 years): 1.2 ± 1.3 Children (>12 years): 0.9 ± 0.4 Adults: 0.8 ± 0.4 Hematuria (score): Children (<10 years): 2.9 ± 1.3 Children (>12 years): 3.0 ± 1.0 Adults: 2.8 ± 1.3	Hypertension presentation (n): Children (<10 years): 0 Children (>12 years): 0 Adults: 5	The accumulation of CD68^+^ macrophages was evident in glomeruli and tubulointerstitium and greater in adults than in the young pediatric group. CD163^+^ is higher in adults (65%) and the older than younger pediatrics (29%). CD204^+^ is higher in adults than in the two groups of pediatrics.	CD68^+^ and CD163^+^ macrophages are related with proteinuria in all groups (r) Children (<10 years)/children (>12 years)/adults CD68^+^ macrophages: 0.71/0.79/0.69 CD163^+^ macrophages: 0.67/0.77/0.73 In children (<10 years) groups the CD68^+^ macrophage is correlated with glomerular hypercellularity (r = 0.86), in children (>12 years) (r = 0.66), CD204^+^ is correlated with it (r = 0.56)	In early phase of IgAN, the alternatively activated macrophages infiltrated the glomeruli and tubulointerstitium, and M2 macrophages were correlated with glomerular matrix expansion.
Zhu G (25)	2006	23–66 (average 42.7)	26/10	Hypercellular glomerulus n: Grade 0 (none)/grade 1 (<25%)/grade 2 (25%–50%): 3/29/4 Mild–moderate glomerulosclerosis n: Grade 0/grade 1/grade 2/grade 3: 3/11/13/9 Tubular atrophy n: Grade 1/grade 2 (<25%):22/14 Tubulointerstitial fibrosis, n: Grade 1/grade 2: 19/17	Median (range) Scr: 130 (91–180) μmol/L eGFR: 66 (40–91) ml/min	Mean proteinuria: 2.3 ± 1.8 g/day 31% of patients had proteinuria > 3 g/day Microscopic hematuria: n = 32 (89%)	Hypertension presentation, n = 16	The number of activated macrophages (27E10) in glomeruli is related with Scr (r^2 = ^0.1) and tubulointerstitium Scr (r^2 = ^0.34). The number of macrophage (Mac387) and CD25^+^ cell in interstitial is related with Scr (r^2 = ^0.33, r^2 = ^0.23) Correlation of the number of tubulointerstitial Mac387 (r^2^ = −0.21) and 27E10 (r^2^ = −0.19) with eGFR	The number of activated macrophages in the glomeruli is weakly related with follow-up Scr (r^2 = ^0.15). Activated macrophage and macrophage in the tubulointerstitium are related with the follow-up Scr (r^2 = ^0.34, r^2 = ^0.33).	The quantities of activated macrophages (27E10) in the glomeruli and tubulointerstitium reflected a poor kidney prognosis.
Utsunomiya Y (26)	1999	Stable group: 30.0 ± 7.9 ESRD group: 30.5 ± 8.5	Stable group: 7/6 ESRD group: 9/5	Mesangial cell cellularity (score) Stable group: 0.8 ± 0.3 ESRD group: 1.4 ± 0.7	NA	Proteinuria (g/day): Stable group: 0.7 ± 0.5 ESRD group: 1.3 ± 0.8	Stable group: Systolic BP: 124.3 ± 22.9 Diastolic BP: 75.2 ± 13.3 ESRD group: Systolic BP: 124.6 ± 21.5 Diastolic BP: 72.6 ± 15.9	CD68^+^ M/C (mesangial localization index) With/without mesangial expression of α-SMA: 1.6 ± 0.3/0.8 ± 0.2 Mesangial expression of α-SMA Stable group/ESRD: 0.3 ± 0.1/1.1 ± 0.3 (*p* < 0.0005) Location: distributed in the mesangial area in IgAN patients with the expression of α-SMA.	Mesangial localization of macrophages is able to drive the phenotypic modulation of mesangial cells correlated with renal damage progression.	Macrophages distributed in the mesangial induced phenotypic modulation of mesangial cells and the expression of α-SMA in mesangial predicted the progressive attenuation in kidney function in IgAN patients.
Arima S (27)	1991	18–66 (average 42.3)	21/24	Controls: n = 8 Mes-Grade 1: n = 20 Mes-Grade 2: n = 14 Mes-Grade 3: n = 11	NA	NA	NA	FMC32^+^ cells (per glomerulus) Controls: 1.32 ± 0.094 Mes-Grade 1: 1.00 ± 0.077 Mes-Grade 2: 2.21 ± 0.153 Mes-Grade 3: 3.85 ± 0.462 FMC32^+^ cells (capillary lumen:Bowman’s:mesangial area) Controls: 100%:0:0, Mes-Grade 1: 52%:39%:9% Mes-Grade 2–3: 40%:40%:19%	The counts of FMC32^+^ cells per glomerulus were correlated with severity of proteinuria (r = 0.387) and the number of cellular crescent (r = 0.779).	The monocyte/macrophages infiltrating the glomeruli were the markers of the high level of mesangial proliferation and glomerular sclerosis. The monocyte/macrophages infiltrating the tubulointerstitium were correlated with tubulointerstitial damage, especially around the sclerosis of glomeruli.
Alexopoulos E (28)	1989	27 ± 14.8	31/3	70% of patients: tubular atrophy and tubulointerstitial fibrosis 38% of patients: small segmental crescents	Pcr (μmol/1): 109.3 ± 64.4 35% of patients: IgM 29% of patients: IgG 91% of patients: C3	Microscopic hematuria Patients: n = 28 Macroscopic hematuria Patients: n = 19 Proteinuria patients: n = 27	Hypertensive patients %: 32.3%	FMC32^+^ macrophages Intraglomerular cells (per glomerular cross-section): Controls (n = 8): 0.9 ± 0.4 IgAN (n = 34): 1.1 ± 0.9 Tubulointerstitial cells (per mm^2^) Controls (n = 8): 75 ± 12 IgAN (n = 34): 278 ± 24	Monocytes/macrophages with plasma creatinine (r = 0.34) T cells and leucocytes in the tubulointerstitium have a positive relationship with tubular atrophy, tubulointerstitial fibrosis, and crescents (*p* < 0.05). T cells and leucocytes in the interstitial with Pcr are positive (*p* < 0.005)	T cells and macrophages played an important role in the pathogenesis of tubulointerstitial lesions and indicated the severity of renal function impairment.
Glomerular										
Kawasaki Y (9)	2009	Favorable group: 11.5 ± 2.7 Unfavorable group: 12.1 ± 3.7	Favorable group: 9/8 Unfavorable group: 4/4	Favorable group: normal urine or minor abnormal urine Unfavorable group: persistent nephropathy	Favorable group/unfavorable group ALB (g/L): First renal biopsy: 42 ± 7/40 ± 6 Second renal biopsy: 44 ± 4/42 ± 3 Scr (μmol/L): First renal biopsy: 48.6 ± 13.3/49.5 ± 11.5 Second renal biopsy: 42.9 ± 9.7/45.1 ± 8.8 Serum IgA (mg/dl): First renal biopsy 262.4 ± 80.3/259.8 ± 77.5 Second renal biopsy 214.1 ± 77.9/277 ± 85.4	Favorable group/unfavorable group Urinary protein excretion (mg/m^2^/h): First renal biopsy Favorable group: 18.6 ± 13.2 Unfavorable group: 20.4 ± 10.6 Second renal biopsy Favorable group: 0.8 ± 1.2 Unfavorable group: 8.3 ± 5.0 Frequency of hematuria (%): First renal biopsy: 100/100 Second renal biopsy: 23.5/100	NA	Favorable group/unfavorable group Glomerular macrophage MPR8^+^ First renal biopsy 6.3 ± 2.2/7.8 ± 3.5 Second renal biopsy 1.7 ± 1.2/5.9 ± 1.6 Glomerular macrophage MPR14^+^ First renal biopsy 1.7 ± 1.4/2.5 ± 1.5 Second renal biopsy 0.4 ± 0.6/0.5 ± 0.5	MPR14^+^ macrophages are the marker of acute-stage inflammation, and MPR8^+^ macrophages are the marker of the chronic stage. MPR8^+^ might reflect a predilection for chronic inflammation and a poor prognosis in children with IgAN.	Renal macrophages expressing MPR8^+^ participated in the progression of sclerotic changes in children with IgAN.
Nagata M (29)	1995	MGA: 12.1 ± 1.1 FGN: 12.7 ± 0.7 DPUN: 12.9 ± 0.9 SCL: 11.8 ± 0.7 Total: 13 ± 0.4	MGA: 5/3 FGN: 11/4 DPUN: 8/5 SCL: 3/2 Total: 27/14	MGA: N = 8 FGN: N = 15 DPGN: N = 13 SCL: N = 5	Serum IgA (mg/dl): MGA: 279 ± 34 FGN: 312 ± 19 DPGN: 247 ± 28 SCL: 243 ± 20	Proteinuria: (g/day) MGA: 0.1 ± 0.04, FGN: 0.6 ± 0.2 DPGN: 1.5 ± 0.4, SCL: 3.1 ± 1.1 U_RBC_ (degree): MGA: 1.4 ± 0.3, FGN: 2.3 ± 0.3 DPGN: 2.4 ± 0.2, SCL: 1.8 ± 0.4 U_WBC_ (HPF): MGA: 1.9 ± 0.4, FGN: 5.7 ± 1.5 DPGN: 8.2 ± 1.9, SCL: 4.0 ± 1.3	NA	ANM/P (glomerulus): MGA: 1.4 ± 0.3 FGN: 5.6 ± 1.3 DPGN: 6.9 ± 0.9 SCL: 0.4 ± 0.3	The relationship of ANM/P with the severity of hematuria and leukocyturia (*p* < 0.01) ANM/GL in MOD and SEV is higher than MIN or SCL (*p* < 0.05). SEV is higher than MOD (*p* < 0.05)	During an active site of glomerular inflammation, macrophages that infiltrated the glomeruli take part in mesangial hypercellularity and are involved in the process of par mesangial destructive lesions, decline of GBM, or injury of the integrity of podocyte.
Tubulointerstitium										
Wang Q (22)	2018	IgAN: 31.8 ± 11.6 Normal control: 32.9 ± 8.5	IgAN: 36/35 Normal control: 3/3	Normal control/IgAN (n): M0: 5/49 S0: 6/54 E0: 6/59 T0: 6/41 T1: 0/9 Tubular interstitial inflammation (1/2/3/4): Normal control: 6/0, IgAN: 30/21/14/6	NA	Proteinuria g/24 h: Normal control: 0.102 ± 0.41 IgAN patients: 3.6 ± 1.9 sEH +: 1.5 ± 1.04 sEH ++: 2.9 ± 1.7 sEH +++: 4.1 ± 3.9 sEH ++++: 5.4 ± 2.6	MAP IgAN: 109.7 ± 9.4 Normal control: 90.4 ± 9.2	IgAN patients have a greater number of CD68^+^ macrophage cells and higher sEH (enzyme) than control people. sEH facilitated M1 polarization via NF-κB pathway activation, and EET accelerated M2 polarization via the activation of the PI3K pathway.	Tubular atrophy and tubulointerstitial fibrosis are correlated with kidney tubular sEH expression degree R = 0.221 Tubular interstitial inflammation is correlated with renal tubular sEH expression levels R = 0.247, *p* = 0.038. sEH is positively related with proteinuria R = 0.452.	sEH promotes M1 polarization, which is related to inflammation and fibrosis. IgAN patients have higher levels of sEH.
Gutiérrez E (30)	2012	Complete recovery: 45.48 ± 19.5 Incomplete recovery: 64.38 ± 12.7	Complete recovery: 13/3 Incomplete recovery: 12/5	Complete recovery/incomplete recovery Mesangial proliferation: 71% (+), 21% (++), 8% (+++)/42% (+), 58% (++) % GS:5.7 ± 9.4/15.5 ± 25.3 % Glomeruli with crescents: 3.8 ± 9.4/5.1 ± 9.6 % Tubules with RBC casts: 41.1 ± 19.2/55.3 ± 17.9 Tubular necrosis: 8% (−), 57% (+), 35% (++)/8% (+), 75% (++) 17% (+++) Interstitial fibrosis: 29% (−), 71% (+)/21% (−), 58% (+), 21% (++)	Complete recovery/incomplete recovery Scr (mg/dl) Initial: 2.9 ± 2.1/4.7 ± 1.9 Peak: 3.6 ± 2.1/7.1 ± 3.2 6 months post-AKI: 1.2 ± 0.9/2.4 ± 1.3 eGFR Initial: 33.7 ± 19.9/15.9 ± 10 Peak: 25.4 ± 15.0 6 months post-AKI: 76.5 ± 27.4/32.0 ± 12.8	Complete recovery/Incomplete recovery Proteinuria (g/day): Initial (at admission): 1.8 ± 1.6/3.2 ± 2.7 Peak: 1.9 ± 1.6/4.9 ± 4.4 6 months post-AKI: 0.4 ± 0.4/1.6 ± 2.2	Complete recovery Systolic BP: 127.5 ± 22.5 Diastolic BP: 73.9 ± 9.8 Incomplete recovery Systolic BP: 125.3 ± 12.6 Diastolic BP: 72.8 ± 11.7	Macrophage-positive area, %/CD163^+^ macrophage score (ρ, *p*) % GS: (0.44, 0.02)/(0.24, 0.22) % Glomeruli with crescents: (0.17, 0.41)/(0.18, 0.36) % Tubules with RBC casts: (0.39, 0.05)/(0.48, 0.01) Tubular necrosis: (0.42, 0.03)/(0.54, 0.003) Interstitial fibrosis: (0.33, 0.09)/(0.29, 0.13) eGFR 6 months post-AKI: (−0.66, 0.001)/(−0.72, 0.001) Proteinuria 6 months post-AKI: (0.08, 0.69)/(0.50, 0.01)	The CD163^+^ macrophage is activated by Hb and ROS; therefore, some anti-inflammation reactions are activated. The CD163^+^ macrophage is related with tubular necrosis and higher proteinuria.	CD163^+^ macrophages can predict the patients with IgAN at high risk for worse renal outcomes with macroscopic hematuria-related acute kidney injury.
Silva GE (14)	2012	Favorable course: 27.7 ± 9.2 Unfavorable course: 27.2 ± 13.0	Favorable course: 10/18 Unfavorable course: 22/12	NA	Scr (mg/dl): At the time of biopsy: Favorable course: 1.4 ± 0.5 Unfavorable course: 1.9 ± 0.4 At the end of follow-up: Favorable course (n = 28): 2.2 ± 0.7 Unfavorable course (n = 34): 3.8 ± 1.3	Proteinuria g/24 h: At the time of biopsy: Favorable course: 1.9 ± 0.8 Unfavorable course: 2.9 ± 1.0 At the end of follow-up: Favorable course: 1.1 ± 0.8 Unfavorable course: 1.0 ± 0.5	NA	Unfavorable courses have more macrophages than favorable courses.	R1: the time of biopsy; R2: the end of the follow-up period Tubulointerstitial macrophages are related with Scr (R1 = 0.28, R2 = 0.46) and proteinuria (R1 = 0.44, R2 = 33), tubulointerstitial α-SMA and NF-kB expression (*p* = 0.01), tubulointerstitial lesions (*p* < 0.01), eGFR (r = −0.531), and renal function (*p* < 0.01)	CD68^+^ macrophages were positively related with the Scr, 24-h proteinuria, process of renal disease, and a worse outcome. The number of macrophages that increased in the tubulointerstitium was regarded as the predictor for poor prognosis in IgAN patients.
Blood										
Esteve Cols C (31)	2020	50.63 (mean age)	14/8	IgAN n (%): M1: 22 (100%) S1: 12 (54.54%) E1: 4 (18.18%) T1-2: 7 (31.82%) Group 1 (n = 13)/Group 2 (n = 9) M1: 13/9 S1: 10/2 (*p* = 0.0274) E1: 2/2 T1-2: 7/1 (*p* = 0.0669)	Scr (mg/dl): IgAN: 1.76 ± 3.03 Group 1 (patients with CD89^+^ MFI < 4000): 2.198 ± 0.389 Group 2 (patients with CD89^+^ MFI > 4000): 1.157 ± 0.193	Proteinuria IgAN: 1.21 ± 1.81 (g/g creatinine) Group 1: 1049 ± 297.7 mg/L Group 2: 785.4 ± 368.6 mg/L IgAN: Hematuria: 7 ± 15.90	NA	In group 1, patients had poor kidney function with higher Scr (*p* = 0.0440), lower eGFR (*p* = 0.0506), and a more severe renal biopsy, with more severe S1 (*p* = 0.0274) and T1-2 (*p* = 0.0669).	CD89^+^ on non-classical monocyte can be seen as the marker of diagnosis in IgAN. Low CD89^+^ monocytes have more severe pathology (S1 and T1-2) and clinical symptoms.	Low expression of CD89^+^ on monocytes, with higher serum degree of Gd-IgA1 and percentages of inflammatory leukocyte subclasses, increased the sensitivity of diagnosis and prognosis in IgAN.

M, male; Scr, serum creatinine; ALB, serum albumin; eGFR, estimated glomerular filtration rate; BUN, urea nitrogen; AOPP, advanced oxidation protein product; ANM/P, average number of glomerular macrophages per patient; MAP, mean arterial pressure; Hb, hemoglobin; ROS, reactive oxygen species; NA, not available; MGA, minor glomerular abnormality; FGN, focal proliferative glomerulonephritis; DPGN, diffuse proliferative glomerulonephritis; SCL, sclerosis; sEH, soluble epoxide hydrolase; EET, epoxyeicosatrienoic acids.

One article illustrated that activated macrophages can be classified into two subclasses, MPR14^+^ and MPR8^+^ macrophages, whose functions were similar to those of M1 macrophages and M2 macrophages (25). MPR14^+^ macrophages are involved in the acute inflammatory response, while MPR8^+^ macrophages are functionally like M2 macrophages and participate in chronic inflammation (9). Yang et al. revealed that the quantities of M0 (CD68^+^), M1 (CD80^+^), and M2 (CD163^+^) macrophages in the kidney tissues of IgAN patients were obviously increased, but the infiltration of M2 macrophages was mostly found in the samples of these IgAN patients (24), which suggested that the polarization of macrophages is mainly to the M2 phenotype. M2 macrophages have a positive correlation with serum creatinine (Scr), 24-h proteinuria, and fibrotic area. However, M0 and M1 macrophages do not have this correlation, which indicate that M2 macrophages are critical in the process of IgAN fibrosis; also, M0 macrophages in the glomeruli can be regarded as the marker of mesangial and endocapillary hypercellularity (32); the count of M0 macrophages per glomerulus is associated with C, M, and final MEST-C sum positively (12). Furthermore, the different subpopulations of M2 macrophage concerning M2a (CD206^+^), M2b (CD86^+^), and M2c (CD163^+^) have distinctive properties when participating in the progression of IgAN. Both M2b and M2c macrophages closely link to percentage of crescents, decline of eGFR, and degree of proteinuria, which suggest that M2 macrophages take part in AKI of glomerular disease with crescents (33), as shown in **Figure 1**.

In IgAN, glomerular injury is also associated with macrophage-mediated immune response. They can stimulate complement system activation (34). M1 macrophages can produce more C3 whose intensity is correlated with the levels of advanced oxidation protein products (AOPPs) in serum than M2 macrophages (12). The AOPPs are associated with the process of IgAN and urine protein, so we can assume that M1 macrophages can be significant by promoting the immune and inflammatory response during the progression in IgAN patients.

### 3.2 The pathogenesis of mononuclear phagocyte system in IgAN

**Figure 2:**
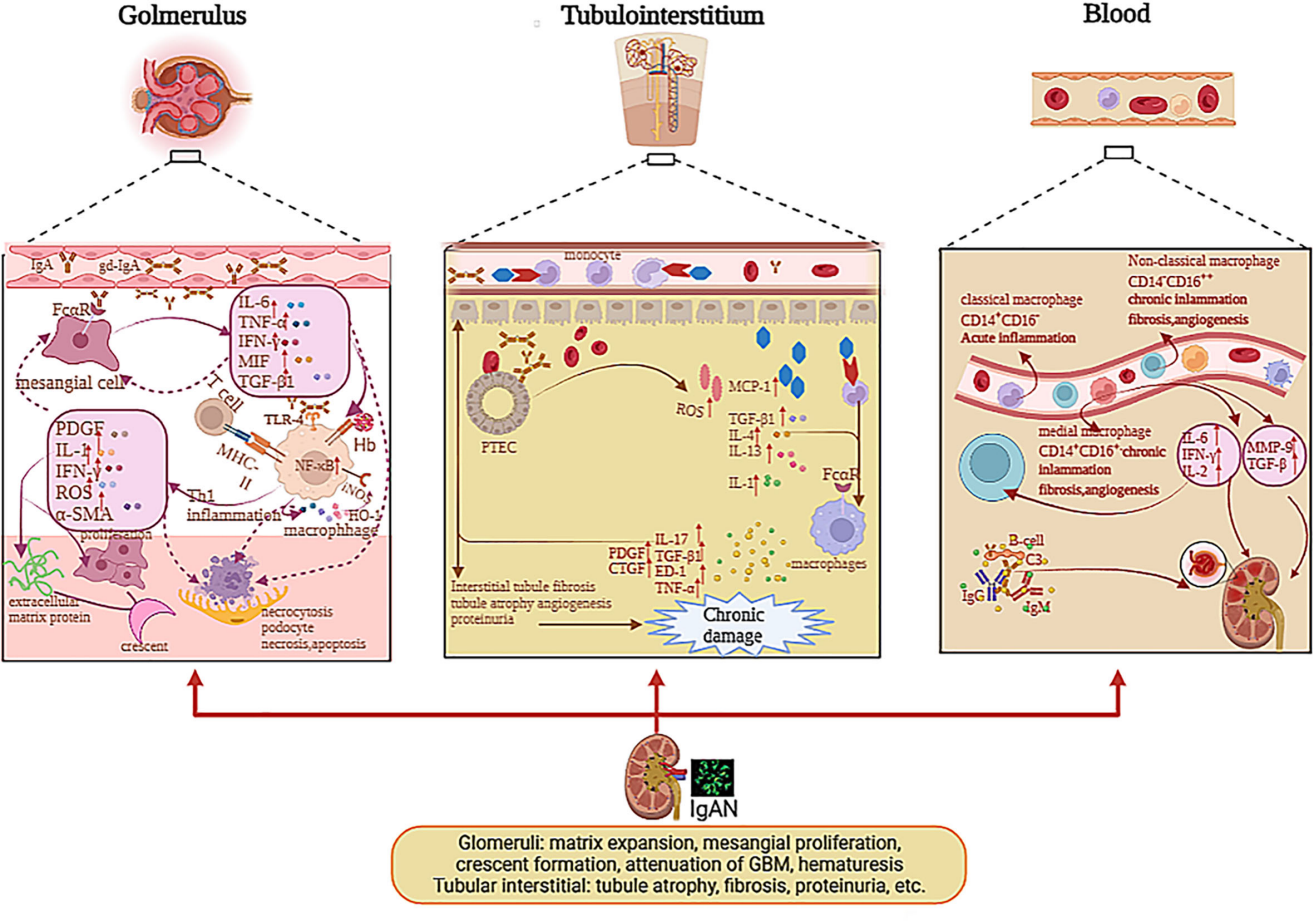
The pathogenesis of mononuclear phagocyte system in glomerulus, tubulointerstitium, and blood with IgAN. The deposition of Gd-IgA1 and relative immune complex stimulate mesangial cells via Fc receptor; therefore, mesangial cells secrete cytokines, which can induce macrophage infiltration and immune cell activation in glomeruli and stimulate themselves. IL-1, TNF-α, and MIF activated NF-κB pathway and affected cell growth, apoptosis, and inflammatory response. The deposition of Gd-IgA1 immune complexes and cytokines release of mesangial cells stimulate the macrophages to activate; hence, macrophages secrete IL-1, IFN-γ, α-SMA, etc., which can lead to mesangial proliferation, extracellular matrix protein, par mesangial destructive lesions with segmental lesions, endocapillary proliferative lesions, crescent formation, and macroscopic hematuria. The macrophages mediated Hb uptake and induced anti-inflammatory pathways by HO-1 synthesis participating in the destruction of the integrity of podocytes and through the mesangio-podocytic-tubular crosstalk; the kidney tubules are damaged via various cytokines and chemokines. In tubulointerstitium, the IgA1 immune complexes and Hb can stimulate PTECs to release ROS, MCP-1, IL-1, etc., which prompt macrophages to infiltrate tubulointerstitium and secrete cytokines, which can facilitate tubulointerstitial fibrosis, tubule atrophy, proteinuria, and angiogenesis, causing renal function loss. In blood, the monocytes were divided into various subclasses including non-classical monocytes (CD14^−^CD16^++^), classical monocytes (CD14^+^CD16^−^), and medial monocytes (CD14^+^CD16^+^). Monocytes can release various cytokines in blood, which can contribute to renal injury in IgAN.

Although the infiltration of macrophages is commonly investigated in IgAN, the mechanism causing this phenomenon is unclear. Some studies suggested that the renal injury of patients with IgAN mainly contributed to the deposition of Gd-IgA1 and relative immune complex and activated complements (2, 4). These sediments can stimulate mesangial cells and macrophages through the C3bi receptor (C3biR) or Fc receptor or MHC class II antigens (35). Ootaka et al. classified the IgAN into two immunohistology types: type C with predominant deposition of complement and type F with dominant glomerular deposits of fibrin-related antigens (FRAs). They interacted with ICAM1/LFA1 to induce macrophage infiltration and immune cell activation in the glomeruli. In type F, activating macrophages are caused by their Fc receptor or MHC class II antigens or the T-cell/macrophage interaction. In type C, a complement-mediated mechanism through the C3bi receptor can activate macrophages and induce proteinuria (35, 36), as indicated in **Figure 2**.

Some researchers thought that IgA1 with under glycosylation, which deposited mesangial cells in the glomeruli with very intimate connection, can induce profibrotic cytokine production and mesangial cell proliferation in patients with IgAN (37), such as monocyte chemotactic protein-1 (MCP-1), platelet-derived growth factor (PDGF), macrophage migration inhibitor factor (MIF), TGF-β, and α-SMA (37, 38). These cytokines can promote M1 macrophages to infiltrate the glomerulus. MIF released by M1 macrophages can increase NF-κB expression via the antagonism of glucocorticoids during acute inflammation (39). In IgAN, MIF expression in proliferative glomerulonephritis is paralleled to the infiltration of macrophages and T cells, as well as associated with the severity of kidney dysfunction, tissue injury, and leukocytic infiltration, but not with the degree of proteinuria (40, 41), as demonstrated in **Figure 2**. The MIF in serum is not significantly different between the healthy group and the trial group, while the MIF in urine and kidney biopsies is higher in the trial group than healthy group (41). IL-1, TNF-α, and MIF activate the NF-κB pathway and affect cell differentiation, programmed death, and inflammatory reaction in IgAN (42). As we all know, NF-κB regulates gene transcription by binding to response elements, which are specific sequences of nuclear DNA (43). Previous studies have revealed the role of NF-kB in the activation of leukocyte cells and the relationship between the activation of NF-κB and macrophages, which migrate to the tubulointerstitium in patients with IgAN (44, 45). Both the deposition of *O*-galactosylated IgA1 immune complexes and cytokine release of mesangial cells can stimulate the macrophages to be activated. At the early stage, M1 macrophages can release multiple cytokines such as IL-1, IFN-γ, IL-6, and α-SMA (1, 2), which support neutrophils to kill the pathogen and recruit other immune cells to secrete chemokine, which can also facilitate glomerulus apoptosis and necrosis.

The macrophages that infiltrate mesangial areas also take part in mesangial proliferation, mesangial destructive lesions with resultant segmental lesions, and endocapillary proliferative lesions; also, the activated macrophages mainly infiltrate glomeruli in the early phase of IgAN. The M1 macrophages can secrete some factors such as PDGF and proinflammatory factor IL-6, which can accelerate regeneration in the early phases of kidney injury. Nagata M. speculated that macrophages might participate in the development of sclerosis by activating the phenotypic variation in mesangial cells by comparing ANM/P with proliferative lesions (29). The phenotypic changes in mesangial cells can release the α-SMA that prompts fibrosis and sclerosis. Mesangial α-SMA expression predicts the process of descent in kidney function in IgAN patients (26, 29), as displayed in **Figure 2**.

Moreover, many apoptotic and necrotic cells can stimulate the M2 macrophages to be activated, which can promote fibroblast proliferation and fibrogenesis at the later phage by secreting a great number of TGF-β and additional profibrotic cytokines. The M2 macrophages also associate with glomerular matrix expansion but not with glomerular hyperplasia (13), which release apoptosis inhibitors of macrophage (AIM) and TGF-β1 that are involved in the IgAN pathogenesis (37). AIM, also called CD5L, is a protein secreted by macrophages, which has a vital function in the immune inflammatory response (46). Recent studies revealed its role in renal fibrosis. For example, in chronic kidney disease (CKD) patients, the area of AIM and macrophage infiltration has a positive correlation with proteinuria and eGFR decrease severity, which indicates that AIM has effects of aggravation in renal damage (46, 47). In IgAN, the expression of AIM has a positive correlation with M2 macrophages, TGF-β1, and the seriousness of kidney fibrosis. TGF-β1 is an important cytokine that promotes renal tubulointerstitial fibrosis (24). The mechanisms of TGF-β1 in renal fibrosis are that it can promote the synthesis of extracellular matrix, cause the decreased degradation of extracellular matrix, and facilitate mesangial cell proliferation by promoting inflammatory response, secreting collagen, and damaging the epithelial and podocyte cells. TGF-β1 can also accelerate myofibroblast trans-differentiation and proliferation (21, 24). One study indicated that macrophages in the glomeruli participated in the occurrence and progression of IgAN by secreting the procoagulant tissue factor, which leads to an extrinsic coagulation pathway, contributing to the deposition of fibrin (28). Above all, we can assume that M2 macrophage is associated with the fibrosis process of IgAN as a kind of profibrotic macrophage. They also play vital roles in the formation of crescents.

With renal impairment, gross hematuria will occur in IgAN patients. Gross hematuria is related with AKI in IgAN (48). There is a higher presence of CD163^+^ macrophages and molecules associated with oxidative stress in samples of subjects with gross hematuria, which means a worse renal outcome. The CD163^+^ macrophages mediate hemoglobin (Hb) elimination, which decreases Hb content and induces anti-inflammatory pathways via increasing the composition of heme oxygenase-1 (HO-1) (49). Gutiérrez E et al. found that CD163 expression and oxidative stress were important prognostic factors for incomplete recovery of kidney function in patients with gross hematuria-associated AKI in IgAN (30, 49).

All in all, we can hypothesize that M1 macrophage is involved in acute inflammatory response at the early stage, which not only can clear the pathogen but also cause apoptosis and necrosis in normal histiocytes. The apoptosis of cells in the glomeruli can stimulate the M2 macrophages to be activated in the later phase, which can secrete some growth and fibrotic factors, which can promote renal sclerosis and fibrosis. Macrophages also can participate in the immune response by activating the T cell as an antigen-presenting cell, leading to cell necrosis, mesangial cell proliferation, extracellular matrix dilatation, and crescent formation. Macrophages that infiltrate the glomeruli can be involved in the progress of par mesangial destructive injury or decline of glomerular basement membrane (GBM) (17) and the destruction of the completeness of podocyte. The podocyte is damaged by various inflammatory cascades, immune responses, and oxidative stress. Through the mesangio-podocytic-tubular crosstalk, the kidney tubules are damaged by various cytokines and chemokines (50).

## 4 The role of mononuclear phagocyte system in tubulointerstitium with IgAN

### 4.1 The relationship between different subtypes of macrophages and clinical characteristics

Tubulointerstitial fibrosis is strong evidence of future progression in IgAN (51). The 13 articles about the macrophages infiltrating the tubulointerstitium in IgAN found that when the macrophages infiltrated the tubulointerstitium, the patients had proteinuria, lower eGFR, tubule atrophy, and tubule interstitial sclerosis (12, 52). Silva GE et al. suggested that the M0 macrophages were positively related with the Scr, 24-h urinary protein, progression of renal injury, and worse outcomes. The degree of macrophages infiltrating the tubulointerstitium can predict the prognosis in IgAN patients (14). Hu W et al. have shown that M2a macrophage counts in the tubulointerstitium were related with Scr (r = 0.363), 24-h urinary protein (r = 0.329), degree of lesion area sclerosis (r = 0.457), and tubulointerstitial fibrosis (r = 0.327). They also found that the number of M2a and M2b macrophages was more than that of M2c in the nephridial histology of patients with IgAN (20). M2a macrophages have a positive relationship with kidney dysfunction, sclerosis of the lesion area, and tubulointerstitial fibrosis, but the relationship between M2c macrophages and glomerular sclerosis and tubulointerstitial fibrosis is negative. M2a macrophages, which mainly distribute in the tubulointerstitium, connect to M1, S1, and T1 among Oxford MEST-C and Lee grade IV–V. The number of M2c macrophages is paralleled to T0, G0, and Lee grade II–III, as exhibited in **Figure 1**. We can hypothesize that macrophages infiltrating the tubulointerstitium are related to the fibrosis of the tubule. Tubulointerstitial fibrosis is a common symptom in the development of chronic kidney disease (53).

### 4.2 The pathogenesis of mononuclear phagocyte system in IgAN

With kidney injury, more macrophages infiltrate the tubule. The macrophages can interact with proximal tubular epithelial cells (PTECs) and collecting duct epithelial cells (CDECs) via FcαR (54). Then, more MCP-1 is released by PTECs (55). A study has indicated that the soluble epoxide hydrolase (sEH) can metabolize the epoxyeicosatrienoic acids (EETs), which have various biological functions, such as vasodilation anti-inflammatory reaction. Wang Q et al. showed that sEH was upregulated in proximal convoluted tubular epithelial cells in IgAN patients (22). Therefore, in the tubule, sEH was correlated with the progression of IgAN. In the tubulointerstitium, macrophages can be involved in inflammatory damage and renal repair, according to the phenotypes of macrophages. M1 macrophages can enhance the inflammatory response, and M2 macrophages prompt anti-inflammation and revascularization (56–58). From the research of Li B et al., we found that CD4 memory T cells and M2 macrophages were in great proportion in the tubulointerstitium (50). With more M2 macrophages infiltrating the peritubular space, the progression of tubulointerstitial fibrosis strongly deteriorates. Indeed, M2 macrophages that infiltrate the tubulointerstitium represent the source of profibrotic factors, concerning TGF-β, endothelin-1, and TNF-α, which induce the degree of matrix composition by permanent parenchyma cells and can secrete the inhibitors of matrix-degrading proteases, including tissue inhibitor of metalloproteinase-1 and plasminogen activator inhibitor (59). Several studies supported that M2 macrophages can enhance the progression of fibrous injury by increasing TGF-β and connective tissue growth factor (CTGF). M2 macrophages localized in areas of renal fibrosis among the patients with IgAN (13). These inflammatory factors can accelerate tubule epithelial cell necrosis, interstitial fibrosis, and tubule sclerosis. This means macrophages are related with tubule fibrosis, which can cause renal function loss, as displayed in **Figure 2**.

Gross hematuria-associated AKI is secondary to acute tubular necrosis, which is facilitated through the Hb secretion from intratubular red blood cell (RBC) casts (60). In the tubulointerstitium, some cases of IgAN with incomplete recovery of kidney function showed a supernal level of ferrous deposition in tubules filled by red blood cell casts, which were correlated with higher oxidative stress by p22-NADPH that deteriorated the acute tubular damage (30, 61).

## 5 The role of mononuclear phagocyte system in blood for the pathogenesis and clinical characteristics

Despite that many articles have described that the macrophages infiltrated the glomerulus and tubulointerstitium, little is known about the relationship between the activation of peripheral blood monocytes and kidney injuries in IgAN. We concluded the association between clinical and pathological characteristics of IgAN patients and infiltration of mononuclear in the blood. In one article, M1 accounted for 100% of the Oxford MEST-C with 22 IgAN patients (31), and they all had proteinuria, hematuria, low CD89^+^ monocytes (mean fluorescence intensity <4,000), a more severe renal biopsy (S1 and T1-2) and clinical manifestation. The expression of CD89 on non-classical monocytes with reductive levels represents a worse kidney prognosis in IgAN patients. Arima S et al. suggested that monocytes in the blood played a significant role in the pathogenesis of mesangial proliferation, non-reversible glomerular injury, and tubulointerstitial tissue damage in IgAN (27).

In the blood, the monocytes are divided into various subclasses across their surface markers including non-classical monocytes (CD14^−^CD16^++^), classical monocytes (CD14^+^CD16^−^), and medial monocytes (CD14^+^CD16^+^). Classical monocytes are correlated with acute tissue inflammation, and the other two types are associated with chronic inflammation, angiogenesis, and fibrosis (31), as shown in **Figure 2**. Monocytes in the blood can clean the serum IgA via FcαR (62, 63), and monocytes preferentially express FcαR transcript in IgAN (64). Peripheral blood monocytes can release metalloproteinases (MMPs), mainly MMP-9, which plays a pivotal role in inflammatory processes in IgAN (65). Enhanced expression of the proto-oncogene, including c-fos and c-jun, can contribute to the progression of IgAN (66). The induction of MMP-9 is related with facilitated c-fos and c-jun mRNA expression in IgAN (67). The monocytes also enhance the expression of endothelin-1 mRNA expression, which was related with the progression of IgAN positively (68). Mononuclear cells secrete high levels of cytokines including IL-2, IFN, and IL-6, which can impact the function of lymphocytes and inflammation (69). Therefore, we can diagnose IgAN patients non-invasively by analyzing the subtype of monocytes and the level of expression of CD89 on non-classical monocytes, and also cytokines such as endothelin-1 in the peripheral blood.

## 6 The role of mononuclear phagocyte system for clinical characteristics and various therapeutic schedules

**Table 1** shows that the infiltrating macrophages in the tubulointerstitium are positively related with Scr, 24-h proteinuria, and decline of eGFR in IgAN (11, 12, 24), and the main subtype in the tubulointerstitium is M2 macrophage (13, 18, 20, 24). Li J et al. described that CD163^+^ macrophages in the tubulointerstitium were paralleled to the eGFR reversely (r = −0.718) and proteinuria (r = 0.520) (18). M2a macrophages, which principally migrate to the tubulointerstitium, are associated with M1, S1, and T1 according to the Oxford MEST-C and Lee grade IV–V in pathology. However, in the glomeruli, M2c macrophages are pertinent to T0, G0, Lee grade II–III (20), and crescents (r = 0.730) (18). Ikezumi Y et al. stated that M0 macrophages were correlated with glomerular hypercellularity (r = 0.86), as well as CD204^+^ macrophages (r = 0.56) (13). The ANM/P in the glomeruli is linked with the degree of hematuria and leukocyturia positively (*p* < 0.001) (29).

Depending on the clinical manifestations and renal biopsy, relevant treatment options can be taken. Soares et al. demonstrated that endocapillary hyperplasia reflected glomerular inflammatory response and that the quantities of CD68^+^ macrophages were a strong sign of E score (32). Patients whose renal biopsies showed crescents or endocapillary hypercellularity (E) were more likely to accept steroids/immunosuppressive (IS) therapy (70–73). Glomerular macrophages including CD206^+^ and CD68^+^ cells show the manifestation for predicting the possibility of reaction to immunosuppression used in patients with high risk of disease progression, and persistent proteinuria exceeded 1 g/day on the basis of Oxford MEST-C score and clinical data (11). Xie D et al. suggested that a high level of CD206^+^ macrophage infiltration in the glomerular is linked to a 40-fold increased probability of immunosuppressive response compared with having lower levels. IgAN patients with a higher number of CD68^+^ macrophages infiltrating in the glomerular had a 13-fold increase in the possibility of reaction to immunosuppression (11), while corticosteroid therapy is particularly effective for IgAN patients whose eGFR are less than 60 ml/min per 1.73 m^2^. The renal injuries of IgAN are associated with inflammatory reactions that macrophages participate in, and controlling kidney inflammation and facilitating epithelial/vascular repair are perhaps the optimal treatment to target renal fibrosis and preserve renal function (74, 75). MCP-1, which is secreted by mesangial cells and PTECs, can induce macrophages to infiltrate the glomerulus as well as the tubulointerstitium (37, 38, 55). Angiotensin II is able to prompt MCP-1 production via the activation of NF-κB (76); therefore, treatment with angiotensin-converting enzyme inhibitors (ACEIs) or angiotensin II type 1 receptor blockers in IgAN can inhibit macrophage infiltration of the glomerulus and tubulointerstitium (77–81).

Some research found that mizoribine, which is a kind of immunosuppressive drug, can alleviate interstitial fibrosis by preventing activated macrophage accumulation (82). Ikezumi Y et al. illustrated that mizoribine stopped the development of renal fibrosis by decreasing the quantities of CD163^+^ macrophage and can inhibit the expression of CD300E on macrophages, which impedes monocyte apoptosis, by comparing the two groups. One group was treated with prednisolone alone (P group; n = 42), the other group was treated with prednisolone plus mizoribine (PM group; n = 48) for a 2-year stage, and patients belonging to the PM group were treated with mizoribine, with a dose of 5 mg/kg per day and not exceeding 150 mg/day within 1 year. From this retrospective study, we can learn that mizoribine can restrain the expression of profibrotic molecules on macrophage induced by steroids and reduce chronic lesions such as segmental or sclerosis in the glomeruli and interstitial fibrosis (*p* < 0.01) when compared to the P group in IgAN (83).

## 7 Conclusion

M1 macrophage is regarded as an acute inflammatory cell participating in proinflammatory reaction in the acute phase during the first 48 h after AKI, while M2 macrophage is relevant to chronic inflammation, which can facilitate fibrosis and angiogenesis in IgAN. M2 macrophages in the tubulointerstitium play a key role in chronic inflammation, which can indicate the chronic process of IgAN patients, while they are pertinent to glomerular matrix expansion in the glomeruli; in this case, we can use mizoribine to halt the fibrosis of kidney and slow down the chronic process. M0 macrophages are relevant to glomerular hypercellularity and crescent formation; according to this condition, patients can be treated with steroids. Further understanding of the pathogenesis of IgAN following macrophage infiltration may eventually lead to their use as biomarkers for assessing the progression of IgAN and identifying new therapeutic targets.

## Author contributions

YL conducted data collection and wrote the manuscript. YG conducted data collection. GX was responsible for the idea and paper revision. All authors contributed to the article and approved the submitted version.
